# Melatonin improves rate of monospermic fertilization and early embryo development in a bovine IVF system

**DOI:** 10.1371/journal.pone.0256701

**Published:** 2021-09-02

**Authors:** Juan Carlos Gutiérrez-Añez, Heiko Henning, Andrea Lucas-Hahn, Ulrich Baulain, Patrick Aldag, Birgit Sieg, Vivian Hensel, Doris Herrmann, Heiner Niemann

**Affiliations:** 1 Institute of Farm Animal Genetics, Friedrich Loeffler Institut (FLI), Mariensee, Germany; 2 Medical-Surgical Department, College of Veterinary Medicine, University of Zulia, Maracaibo, Venezuela; 3 Clinic for Gastroenterology, Hepatology and Endocrinology, Hannover Medical School (MHH), Hannover, Germany; University of Florida, UNITED STATES

## Abstract

The developmental competence of male and female gametes is frequently reduced under *in vitro* conditions, mainly due to oxidative stress during handling. The amino-acid derived hormone melatonin has emerged as a potent non-enzymatic antioxidant in many biological systems. The goal of the present study was to evaluate the effects of melatonin on post-thaw sperm quality, fertilizing ability, and embryo development and competence *in vitro* after *in vitro* fertilization. Frozen-thawed bovine spermatozoa were incubated either in the presence of 10^−11^ M melatonin (MT), or its solvent (ethanol; Sham-Control), or plain Tyrode’s Albumin Lactate Pyruvate medium (TALP, Control). Computer-Assisted Sperm Analysis (CASA) and flow cytometry data after 30 min, 120 min, and 180 min incubation did not reveal any significant effects of melatonin on average motility parameters, sperm subpopulation structure as determined by hierarchical cluster, or on the percentage of viable, acrosome intact sperm, or viable sperm with active mitochondria. Nevertheless, *in vitro* matured cumulus-oocyte-complexes fertilized with spermatozoa which had been preincubated with 10^−11^ M melatonin (MT-Sperm) showed higher (*P* < 0.01) rates of monospermic fertilization, reduced (*P* < 0.05) polyspermy and enhanced (*P* < 0.05) embryo development compared to the Control group. Moreover, the relative abundance of *MAPK13* in the *in vitro*-derived blastocysts was greater (*P* < 0.05) than observed in the Control group. In conclusion, adding melatonin to the sperm-preparation protocol for bovine IVF improved proper fertilization and enhanced embryonic development and competence *in vitro*.

## Introduction

The handling of sperm and oocyte in IVF usually is associated with oxidative stress that leads to a higher production of reactive oxygen species (ROS) during the co-incubation period [1] with potentially detrimental effects on both gametes, ultimately leading to impaired *in vitro* embryo development. The use of specific antioxidants such as melatonin to neutralize ROS could protect gametes against adverse ROS effects and may play a significant role in improving the efficiency of assisted reproductive technologies (ARTs) [2–5]. We have recently shown that melatonin enhanced bovine oocyte competence and embryo development when added during *in vitro* maturation of cumulus-oocyte-complexes from juvenile and adult donors [6].

The potent scavenging molecule melatonin (N-acetyl-5-methoxytryptamine) is critically involved in the circadian rhythm by secretion from the pineal gland, but local melatonin production has been detected in extra-pineal organs such as reproductive tissues [7–9] where it plays an essential role as an anti-oxidative molecule in intracellular regulation of ROS levels. Its accumulation in reproductive fluids such as seminal plasma [10] suggests that it could be critical for male reproductive function, including sperm motility and morphology [11], sperm viability, and fertilizing ability [12]. Nevertheless, recently it has been shown that enhanced adhesiveness of sperm to the zona pellucida and higher fertilization rates induced by melatonin, were not associated with intracellular ROS levels reduction [13].

Fertilization involves a coordinated sequence of molecular events starting with the fusion of sperm and egg, followed by fusion of both pronuclei and concomitantly the maternal and paternal chromosomes [14]. Massive epigenetic reprogramming of the paternal and maternal chromatin during fertilization and early cleavage is initiated by maternal RNAs and proteins accumulated during oogenesis [15]. Nevertheless, the proportion of good quality embryos was significantly lower in infertile males than those with normal fertility [16], suggesting that preimplantation embryo development does not rely exclusively upon maternal regulation.

Knowledge of melatonin function on post-thaw bull semen, specifically during co-incubation of male and female gametes, and its impact on fertilization ability and *in vitro* embryo competence is scarce. Reports showed contradictory results and focused mainly on the anti-oxidative pathway [17, 18]. To date, little is known whether the putative beneficial effects of melatonin on spermatozoa also have impact on early embryo development. Enhancing gametes and embryo competence during *in vitro* embryo production has been a primary goal in ART since it allows increasing efficiency, and decreasing cost. Several studies have shown that melatonin improved embryo development *in vitro*, but insight into on the underlying mechanisms of improved embryo development is largely unknown; an anti-oxidative pathway was suggested [9, 18–22].

The goal of the present study was to evaluate the impact of melatonin in an established bovine IVF-protocol on basic sperm function, their ability to fertilize *in vitro*, and embryo development after IVF. To gain insight into embryo competence, we analyzed a panel of developmentally important genes involved in preimplantation embryo development, including the lineage allocation and cell polarity Cadherin 1 (*CDH1*), the caudal type homeobox 2 (*CDX2*), the regulator of cell proliferation and differentiation MAP kinase activity (*MAPK13*)], the water transport [water transport gene aquaporin 3 (*AQP3*)], the two apoptosis regulators [BCL2 Associated X, Apoptosis Regulator (*BAX*), and Heat shock protein beta-1 (*HSPB1*)], and the pluripotency transcripts [POU domain, class 5, transcription factor 1 (*POU5F1*), and the Nanog Homeobox (*NANOG*)].

## Materials and methods

### Reagents, melatonin dissolving, and stock solutions

Unless otherwise stated, all reagents used in this experiment were provided by Sigma-Aldrich (Steinheim, Germany). The melatonin stock solution was prepared in TALP (Tyrode’s medium base, albumin, lactate and pyruvate) medium through serial dilution in ethanol as previously described [6]. TALP composition as previously describe in Parrish et al. [23, 24], supplemented with 6 mg/ml fatty acid free BSA (FAF-BSA, A7030-10G), 0.05 mg/ml gentamicin, 0.028 mg/ml Na-pyruvate.

Three stock solutions were formed: a standard stock of plain TALP (Control), a stock for sham-treated samples containing 1% ethanol (Sham-Control), and a stock solution containing 1 nM Melatonin (MT). Aliquots of 30 μL from each stock solution were stored at -20°C for no longer than four weeks. Working solutions contained 495 μL TALP medium plus 5 μL of a thawed stock solution to prepare medium for each of the experimental groups (Control, Sham-Control, and MT). The final concentration of melatonin and ethanol in the experiment were 10^−11^ M and 0.01%, respectively. The concentration corresponds to the endogenous melatonin levels in bovine follicular fluid [25]. All working solutions were incubated for one hour at 38.5°C and 5% CO_2_ in a humidified atmosphere prior to experimentation.

### Study design

This study included a set of two major experiments to evaluate the effects of an addition of melatonin to the sperm preparation protocol on sperm function, fertilization ability and embryo competence *in vitro* after use in IVF.

#### Experiment 1 (sperm functionality)

In the first experiment, frozen-thawed semen from four Holstein-Friesian bulls with proven fertility was used. The impact of melatonin on sperm motility, acrosome integrity, and mitochondrial activity in viable sperm was determined by Computer-Assisted Semen Analysis (CASA) and fluorescence-activated cell sorting (FACS), respectively. Three experimental groups were compared by flow cytometry and CASA in conjunction with cluster analysis of single sperm data. Spermatozoa were either regularly processed (Control), processed in presence of the solvent for melatonin, i.e. ethanol, (Sham-Control) or processed in presence of melatonin (MT-Sperm).

#### Experiment 2 (fertilizing ability and embryo development *in vitro*)

In the second experiment, semen from one of the above bulls was selected to unravel putative melatonin effects on *in vitro* fertilization ability and early embryo development. The selected bull had a record of regular IVF fertility (32% blastocyst rate along 20 IVF cycles). Spermatozoa were either treated with melatonin prior to coincubation of gametes using the same experimental groups (Control, Sham-Control, MT-sperm) and one additional group where melatonin was included in the *in vitro* fertilization medium (MT-Coincubation).

### Sperm preparation

Frozen-thawed semen from the same batch was used to avoid the influence of variation amongst ejaculates and cryopreserved straws [26]. Six semen straws in the first and three in the second experiment, each with a concentration of 20–25 x 10^6^ spermatozoa / 0.5-mL, were thawed in a water bath at 33°C for 1 min. Then, each straw was placed over 1.0 mL of a commercial colloidal gradient-solution for selecting and purifying bull spermatozoa (BoviPure™, Nidacon, Mölndal, Sweeden). The Bovipure solution was prepared according to the manufacturer’s instructions with slight modifications. Briefly, two concentrations of the Bovipure solution were prepared in 1.5 mL Eppendorf centrifuge tubes, one at 40% (200 μl BoviPure™ + 300 μl BoviDilute™), and the other at 80% (400 μl BoviPure™ +100 μl BoviDilute™). Prior to use, both colloidal solutions were stabilized for one hour in the incubator at 39°C and with 5% CO_2_ in air. During thawing, the 40% solution was placed over the solution at 80%, and then semen was placed on top of this colloidal solution prior to centrifugation.

After centrifugation at room temperature at 400 *g* for nine minutes, 900 μL supernatant from each sample was removed, leaving the sperm pellet in ~100 μL colloidal solution. Then, the resuspended pellets from one bull were pooled and mixed by pipetting up and down nine times until a pure sperm-suspension was achieved that was subsequently split into aliquots of 100 μl each into three new 1.5 ml Eppendorf tubes. Each sample of purified sperm was then assigned to the Control, Sham-Control or MT-Sperm group and centrifuged for three min at 400 *g* with 500 μl of the corresponding TALP medium. After removing 300 μL supernatant, the remaining sample was homogenized and gently mixed. Then, each sperm suspension (Control, Sham-Control, MT-Sperm) was aliquoted in triplicates of 100 μL and incubated at 39°C and 5% CO_2_, leaving the tube partially lid close allowing the gas mixture enter until evaluation after 30 minutes, 120 minutes, and 180 minutes, respectively.

### Sperm motility and Computer-Assisted Sperm Analysis (CASA)

In order to assess the effects of melatonin on *in vitro* sperm kinematics and motion parameters at different incubation periods, we used the IVOS^®^ II CASA system (version 1.10, Hamilton Thorne, Beverly, USA). Prior to evaluation, the samples were gently homogenized. Then, 10 μL of the sample was placed in a Makler chamber and analyzed. Instrument settings of the CASA system are shown in **S1 Table**.

All measurements were performed in triplicate, each analyzing five capture frames. The mean of the three replicates was used for statistical evaluation of average CASA parameters. The CASA parameters included total motility (%), progressive motility (%), straight line velocity (VSL: μm/s), curvilinear velocity (VCL: μm/s), average path velocity (VAP: μm/s), amplitude of lateral head displacement (ALH: μm), beat cross frequency (BCF: Hz), linearity (LIN: %), straightness (STR: %) and wobble (WOB: %):

### Flow cytometric evaluation of spermatozoa (sperm viability, acrosome integrity, and mitochondrial activity)

Measurements of the fluorescently stained sperm were made using a Gallios™ flow cytometer (Beckman Coulter, Krefeld, Germany), equipped with three lasers for excitation at 405 nm (Violet Solid State Diode, 40 mW), 488 nm (Blue Solid State Diode, 22 mW), and 638 nm (Red Solid State, 25 mW).

In parallel to motility assessments, sperm plasma membrane integrity and acrosome integrity were assessed using combined staining with Sybr-14 / Propidium iodide (PI) from the LIVE/DEAD^®^Sperm Viability Kit (L-7011, Molecular Probes^®^, Goettingen, Germany) and fluorescently-labeled peanut agglutinin (PNA-AF647, Invitrogen™). Additionally, the mitochondrial membrane potential (Mito Tracker™ Deep red FM, Invitrogen™) was evaluated in combination with Sybr14/PI staining to allow to discriminate viable and dead spermatozoa. Stock solutions of Sybr14 (1 mM) were diluted first 1:10 in DMSO kept frozen at– 20°C and then further diluted 1:100 with Ca^2+^-free Phosphate Buffered Saline solution (PBS) (final concentration: 100 μM), stored at 4°C, and used within one week. PI stock solutions (2.4 mM) were diluted 1:10 with Ca^2+^-free PBS (final concentration: 240 μg/mL) and stored at 4°C. Mito Tracker™ Deep Red FM (MitoTracker Deep Red) stock solution (1 mM) was diluted 1:10 in DMSO kept frozen at– 20°C and further diluted 1:100 in Ca^2+^-free PBS as working solution (final concentration: 10 μg/mL). The working solution was stored at 4°C and used within four weeks. The PNA-AF647 stock solution (0.1 mg/mL) was diluted 1:10 in Ca^2+^-free PBS. A dye mix for evaluation of sperm viability and acrosome integrity (Premix 1) was prepared by mixing 5 μL Sybr14, 3 μL PI, and 5 μL of PNA-AF647 (final concentration: 10 μg/mL). The dye mix for evaluating mitochondrial membrane potential in viable sperm (Premix 2) was prepared by mixing 5 μL Sybr14, 3 μL PI, and 1.5 μL of MitoTracker Deep Red. After the designated incubation times, samples for determining sperm viability and acrosome integrity were prepared by adding 10 μL sperm sample to 480 μL preheated at 37°C TRIS solution plus 9.25 μL Premix 1 (Syrb14/PI/PNA-AF647) and incubated in the dark for 15 min at 37°C. Samples for determining sperm viability and mitochondrial membrane potential were prepared by adding 10 μL of each sperm sample to 480 μL of preheated at 37°C TRIS solution plus 13 μL Premix 2 (MitoTracker Deep Red) and incubated in the dark for 15 min at 37°C. Two sub-samples from treatment x times x bull combination were prepared and analyzed as technical repeats. Results of technical repeats were averaged prior to performing the statistical analysis. The flow cytometer was set at a medium flow rate for 60 seconds. Data from 10.000 spermatozoa were obtained for each sample. The machine settings and the compensation matrix are summarized in **S2 Table**. A commercial sheath fluid was used (CytoFlex Sheath Fluid, B51503, Beckman Coulter, Life Sciences, Krefeld, Germany).

The gating strategy was as follows: Forward and side scatter signals were used to discriminate single spermatozoa from debris. Subsequently, the viable sperm population (Sybr14 positive & PI negative) with intact acrosomes (PNA-Alexa negative) was determined. In analogy, the percentage of viable spermatozoa with active mitochondria (MitoTracker Deep Red positive) was quantified. The threshold between high and low mitochondrial membrane potential was defined for samples after 15 min incubation period at 37°C and kept constant throughout the experiment.

Sperm viability, acrosome integrity, and mitochondrial activity were evaluated by determining the percentages of viable sperm, viable sperm with intact acrosomes, viable sperm with acrosome reacted and viable sperm with high mitochondria transmembrane potential (MMP). Viability and acrosome integrity were evaluated with a combined staining of Sybr14, PI, and PNA-AF647. Viable sperm (Sybr14 positive/PI negative) were assessed either as acrosome intact (PNA-AF647 negative) or acrosome defect/reacted spermatozoa (PNA-AF647 positive). The percentage of viable sperm with a high mitochondrial transmembrane potential (high MMP) was determined with a combined staining with Sybr14, PI and MitoTracker Deep Red.

### *In vitro* embryo production

#### *In vitro* maturation (IVM)

Selected cumulus-oocyte complexes (COCs) of grade 1 to 2 quality (homogeneous dark cytoplasm and at least three layers of compact cumulus cells) were collected by aspirating follicles (2–6 mm in size) from postmortem ovaries. Ovaries from Holstein cows were collected at a local slaughterhouse (Westfleisch SCE mbH, Meat Center Lübbecke Rote Mühle 54–56, 32312 Lübbecke), transported to the laboratory into 0.9% NaCl solution containing penicillin and streptomycin, and processed within three hours after collection. COCs were incubated in groups of 25–30 in 250 μL *in vitro* maturation medium at 38.5°C and 5% of CO_2_ in a humidified atmosphere for 24 h into four-well dishes (Nunclon™ Delta Surface, Thermo Scientific, Roskilde, Denmark). The *in vitro* maturation medium contained tissue culture based-medium 199 (TCM, Sigma-Aldrich), enriched with 1 mg/ml fatty acid-free bovine serum albumin (FAF-BSA, A7030-10G, Sigma-Aldrich), and 10 IU/ml equine chorionic gonadotropin (eCG) and 5 IU/ml human chorionic gonadotropin (hCG) (Suigonan^®^, MSD, Intervet, Unterschleissheim, Germany).

#### *In vitro* fertilization (IVF)

After maturation, expanded COCs were divided into four groups and each placed in 250 μL fertilization medium (Fert-TALP) as previously described [23, 24], supplemented with 6 mg/mL of FAF-BSA, 10 μM hypotaurine, 1.0 IU/ml heparin, and 1.0 μM epinephrine. Three experimental groups were fertilized with sperm prepared as described above (Control, Sham-Control, and MT-Sperm) after ~15 min of incubation (the time needed for COCs washing from the IVM medium). In the fourth group, sperm from the Control group were used and melatonin was included only in the IVF medium (MT-Coincubation) to allow differentiation of effects from melatonin on the resulting embryos that originate from the co-incubation period and effects that could be ascribed to the melatonin-treated sperm. Prior to IVF, sperm samples were washed, eluted, and centrifuged for three min at 400 g with 500 μl fertilization medium (Fert-TALP), the supernatant was removed leaving the purified sperm in ~ 100 μL Fert-TALP suspension. Insemination was performed with a ratio of 100,000 sperm cells/100 μL of IVF medium (total 250,000 sperm cells per well containing 250 μL of IVF medium). Semen quality of samples from the selected bull was as follows: Total motility: 71.0±2.0%, Progressive motility: 69.2±1.9%, viable sperm with high mitochondrial membrane potential: 77.7±1.9%, viable sperm with intact acrosome: 42.5±2.1%. All values are from the Control group after 15 minutes incubation. A comparison of all bulls is shown in **S3 Table**.

#### *In vitro* culture (IVC)

After IVF (18–20 h), cumulus cells from the presumptive zygotes were removed by vortex the oocytes in tissue culture medium (TCM) 199 medium with HEPES modification (TCM medium 199, M252, Sigma-Aldrich) at 3,000 rpm for five minutes and placed into four-well dishes containing 500 μL *in vitro* culture (IVC) medium based on synthetic oviductal fluid (SOF) [27, 28], enriched with 4 mg/ml of FAF-BSA-covered with 600 μL mineral oil (GYNEMED, Lensahn, Germany), incubated at 38.5°C, 5% of CO_2_ and 5% of O_2_ in a humidified N_2_, until blastocyst formation (day 7.5). Unlike IVC, both IVM and IVF system were oil-free.

IVF outcomes were evaluated by determining rates of cleavage, blastocysts, and advanced blastocysts. Cleavage rate was determined 72 h post-insemination (hpi), and blastocysts rate at 180 hpi (day 7.5) as the percentage of cleaved zygotes (Control n: 156; Sham-Control: 147; MT-Sperm: 145; MT-Co-incubation: 152). Early and non-expanded blastocysts (mid blastocysts) were graded as blastocysts; while expanded, hatching, and hatched blastocysts as advanced blastocysts. Six replicates of *in vitro* embryo production were performed.

### Determination of fertilization

The fertilization rate was determined by pronuclear (PN) assessment using lacmoid staining according to an established protocol in our lab. A lacmoid stock solution (2.2%) was prepared by dissolving 1.0 g lacmoid powder (CarlROTH^®^, Karlsruhe, Germany) in 45 ml slightly heated acetic acid (ROTIPURAN^®^, 100% p.a., CarlROTH^®^). Then, a working solution (1% Lacmoid in 45% Acetic Acid) was prepared by adding 5.0 ml stock solution to 5.5 ml of H_2_O (Milli-Q^®^).

After 18–20 h IVF, cumulus cells from the presumptive zygotes were removed by vortexing the oocytes in TCM medium at 3,000 rpm for five minutes, followed by washing in phosphate-buffered saline (PBS, Sigma-Aldrich) supplemented with 1% polyvinyl alcohol (PVA, Sigma-Aldrich) (PBS-PVA). Next, 5 to 6 zygotes were mounted into small drops of PBS-PVA (approx. 0.3 μL) on a glass slide between two parallel lines of paraffine and covered with a coverslip. This procedure was performed under a stereomicroscope at room temperature. Afterward, slides were put into fixative solution (one-part of acetic acid: three parts of ethanol 100%) for 24 h until evaluation. Finally, the slides were removed from the fixative solution and the lacmoid working solution gently infused between slide and coverslip with an insulin syringe. The pronuclei were visually inspected by using phase-contrast microscopy (Olympus BH2, Tokyo, Japan) switching different-fold magnifications amongst the objectives.

**Fig 1:**
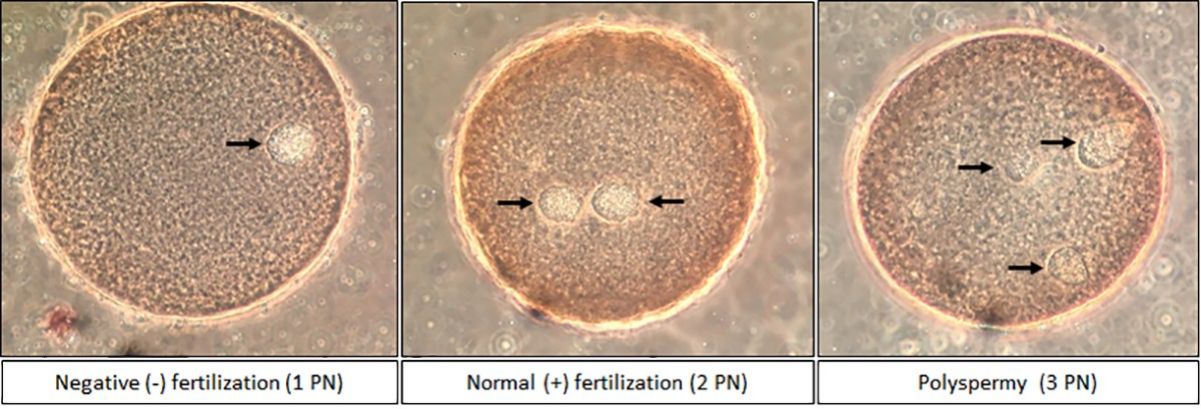
Pronuclear (PN) assessment and determination of fertilization via Lacmoid staining to evaluate the *in vitro* fertilization rate (arrows indicate the pronuclei).

Regular fertilization was defined when two pronuclei (2 PN) were visible. Fertilization failure was diagnosed when either one or no pronuclei were identified. Polyspermy was assumed when three or more pronuclei were identified (**Fig 1**). Zygotes from three different IVF cycles were evaluated (Control n: 56; Sham-Control: 76; MT-Sperm: 74; MT-Co-incubation: 64).

### Gene expression analysis in *in vitro*-derived embryos

Quantification of the relative expression of the developmental genes Cadherin 1 (*CDH1*), caudal type homeobox 2 (*CDX2*), the regulator of MAP kinase activity (*MAPK13*), the water transport gene aquaporin 3 (*AQP3*), the apoptotic regulators BCL2 Associated X, Apoptosis Regulator (*BAX*), and Heat shock protein beta-1 (*HSPB1*), and the pluripotent POU domain, class 5, transcription factor 1 (*POU5F1*), and the Nanog Homeobox (*NANOG*) was performed using the primers listed in **Table 1**.

**Table 1:** Primers sequences used for the real-time qPCR.

Gene	Primer sequences: (5´-3´)	Fragment size/bp	Accession no.
***CDH1***	F: GACACCCGGGACAATGTGTA	86	NM_001002763
	R: GCCCCTATGTAACTGGCTCAA		
***CDX2***	F: CAGAGAGGCAGGTTAAAATTTGGT	80	NM_001206299.1
	R: CTGCTGTTGCAACTTCTTCTTGTT		
***MAPK13***	F: GAAAACGTCATCGGGCTTCTG	189	NM_001014947.1
	R: AGCTGAGTGGATGTACTTAAGACCC		
***AQP3***	F: CTGGGCGCTGGAATTATCTTC	78	NM_001079794
	R: GCCCGAAACAATAAGCTGGTT		
***BAX***	F: GGCTGGACATTGGACTTCCTTC	112	NM_173894.1
	R: TGGTCACTGTCTGCCATGTGG		
***HSPB1***	F: CTGGACGTCAACCACTTC	180	NM_001025569.1
	R: GGACAGAGAGGAGGAGAC		
***POU5F1***	F: AGAAGCTGGAGCCGAACC	85	NM_174580.3
	R: CTGCTTTAGGAGCTTGGCAAA		
***NANOG***	F: TCAGCTACAAGCAGGTGAAGAC	96	NM_001025344.1
	R: GCATGCCATTGCTATTCCTC		
***GAPDH***	CCCACTCCCAACGTGTCTGT	89	NM_001034034.2
	CCTGCTTCACCACCTTCTTGAT		
***Rabbit Globin***	F: TACTTCCCCCACTTCGACTTCA	74	NM_001082389.2
	R: AGGGCTTCGGACACCTTCTT		

F: forward; R: reverse.

To RNA preparation, Poly(A)+ RNA was isolated using the Dynabeads^®^ mRNA Direct Kit (Life Technologies) according to the manufacturer’s instruction with some modifications. Briefly, pools of three expanded blastocysts from each experimental group (Control: *n*: 6, Sham-Control: *n*: 9, MT-Sperm: *n*: 7, MT-Co-incubation: *n*: 7; each was considered as RT-qPCR replicate) derived from six replicates of IVP cycles were thawed in 40 μl of lysis-binding buffer (100 mM Tris-HCL, at pH 8.0, 500 mM LiCl, ten (10) mM EDTA, 1% LiDS, 5.0 mM DTT). Then 3.0 pg rabbit globin mRNA was added as external standard and incubated at room temperature for 10 minutes. Poly(A)+ RNA was isolated using the Dynabeads^®^ mRNA Direct Kit (Life Technologies) according to the manufacturer’s instruction with some modifications. Then 3.0 pg rabbit globin mRNA was added as internal standard and incubated at room temperature for 10 minutes. Prewashed Dynabeads^®^ Oligo d(T)25 (5 μl) were pipetted to the lysate and incubated for 15 minutes at 25°C on a shaker for binding poly(A)+ RNA to the beads. The beads were separated by using a Dynal MPC-E-1 magnetic separator, washed once with washing buffer A (10 mM Tris-HCL. pH 8.0, 0.15 mM LiCl, 1.0 mM EDTA, 0.1% LiDS) and two times with washing buffer B (10 mM Tris-HCL. pH 8.0, 0.15 mM LiCl, 1.0 mM EDTA). The poly(A)+ RNA was eluted from the beads by incubation in 10 μl sterile water at 68°C for two minutes, and the mRNA was used immediately as input for the reverse transcription reaction.

Reverse transcription (RT) was conducted in a 20 μl volume consisting of 4.0 μl of 5x RT buffer (Life Technologies), 1.0 mM dNTP (Life Technologies), 20 Units RNAsin^®^ (Life Technologies), 100 Units M-MLV reverse transcriptase (Life Technologies, Cat.no. 28025–013) and 2.5 μM random hexamer primer (Life Technologies). Afterward, samples were incubated at 25°C for ten minutes for primer annealing and then incubated at 42°C for one hour. Finally, the samples were heated to 90°C for 5 minutes. The cDNA was diluted to a concentration of 0.1 embryo equivalent/μl. Two μl were used for Real-Time PCR amplification.

Finally, Real-time qPCR was carried out in 96-Well Optical Reaction Plates (Life Technologies). The PCR mix in each well included 10 μl of 2x Power SYBR_Green PCR Master Mix (Life Technologies, Cat.no. A25742), and 0.1 μM of each of the forward and reverse primers and 2.0 μl cDNA in a final reaction volume of 20 μl. The PCR reaction was carried out in an ABI 7500 Fast Real-Time System (Applied Biosystems) using the following program: denaturation and activation of the Taq Polymerase during 10 min at 95°C followed by 40 cycles at 95°C during 15 seconds and 60°C for one minute, followed by slow heating for displaying a dissociation curve of the product. Data generated by the Sequence Detection Software 1.4 were transferred to Microsoft Excel for analysis. Differential mRNA expression of each target gene was calculated by the Relative Standard Curve Method. A cDNA dilution from pooled blastocysts mRNA was included on every plate to yield standard curves for each gene. Glyceraldehyde 3-phosphate dehydrogenase (*GAPDH*) as a housekeeping gene expressed in embryos and rabbit globin (as external standard) were used as the internal controls to give a standard curve for each gene as our lab previously reported [29]. Standard curves were used to calculate the relative concentration of each target gene to be normalized to the geometric mean from Globin/*GAPDH* expression results for each sample.

### Statistical analysis

#### General statistics

Data were statistically analyzed using the JMP software (Version 15; SAS Institute, Inc., Cary, NC, 2015) and the Statistical Analysis package (SAS)^®^ (Version 9.4, Cary, NC, USA). Significant differences were defined at *P* < 0.05 except for gene expression analysis where a potential statistical trend was also considered (*P* ≤ 0.10). All data are expressed as LS mean ± SEM (standard error of the mean) unless otherwise stated.

#### Sperm motility assessment

Prior to analysis, a test of normality was assessed through Shapiro Wilk W test and unequal variance through Levine test. To evaluate the effects of treatment and incubation time (30-min, 120-min, and 180-min) on sperm motility and sperm kinetics a linear mixed model fit by Restricted Maximum Likelihood (REML) was applied (JMP). To consider repeated measures, bull was considered as random effect, while treatment and incubation time and their interaction were treated as fixed effects. Tukey multiple pairwise comparisons were used to compare differences between experimental groups.

In addition, a sperm subpopulations analysis (cluster analysis) was performed to identify putative changes in motility patterns due to treatment or incubation time. Cluster analysis considered the data from eight motility descriptors (VSL, VCL, VAP, ALH, BCF, LIN, STR, and WOB) for every single motile sperm (*n* = 153.016). Data from all incubation times and media were combined in one data set. After checking for normality, parameters were correlated (PROC UNIVARIATE and PROC CORR, SAS^®^). Only variables correlating <0.9 (VSL, VCL, VAP, ALH, BCF, LIN, STR, and WOB) were selected for the clustering procedure. Chosen variables were standardized to a mean of 0 and a standard deviation of 1 to avoid bias in the clustering procedure. Then, a hierarchical clustering method was conducted using squared Euclidean distance as distance measurement and the ‘centroid’ algorithm for cluster fusion (PROC CLUSTER, SAS^®^). The choice of a suitable solution was guided by the cubic clustering criterion (CCC), pseudo-F statistics, and pseudo-t^2^ values. A Pearson Chi-square test was used to compare distribution of sperm to the different clusters between time points and treatments (PROC FREQ, SAS^®^). Cramer’s V (ranging from 0 to 1) was used, to evaluate effects from factors treatment or time. Results were interpreted in analogy to the proposed guidelines (Cohen, 1988): V < 0.10 = no effect, 0.10 < V ≤ 0.30 = slight effect, 0.30 < V ≤ 0.50 = moderate effect, V > 0.50 = large effect.

#### Fertilization test and IVF outcomes

To analyze the effect of treatment on the distribution of regularly fertilized and polyspermic oocyte penetration a Generalized Linear Model (GLM) from JMP (SAS^®^) with Chi-Square statistic test was used considering a Poisson distribution model of fertilization and sperm-egg interaction *in vitro* as previously suggested [30]. The other categorical data from IVF outcomes and embryo developmental rates (e.g., cleavage, blastocysts, and advanced blastocysts) were compared using the logistic procedure (PROC LOGISTIC, SAS^®^). Analysis of maximum likelihood estimates was performed to determine the odds ratio estimates, the confidence interval limits for relative risks of embryo developmental rates through the logistic procedure from SAS^®^.

#### Gene expression in *in vitro*-derived expanded blastocysts

The influence of treatment on gene expression was analyzed using a Generalized Linear Model (GLM) from JMP (SAS^®^). Test of normality was assessed through Shapiro Wilk W test and unequal variance through Levine test. All data are expressed as the LS mean values for each set of data ± SEM.

## Results

### Sperm motility

The effects of treatment on sperm motility and CASA parameters are presented in **Table 2**. The overall means for the three different experimental groups (Control, Sham-Control, MT-Sperm) show that significant effects of melatonin on overall motility or sperm kinematics were not evident. The LS means for MT-Sperm were higher for total motility, progressive motility, VCL, and VSL when compared to the Control group (*P* < 0.05), but did not differ from Sham-Control samples (*P* > 0.05).

**Table 2:** Effects of melatonin addition (all time points combined: 30 min, 120 min, and 180 min) to the IVF protocol on sperm motion parameters assessed via CASA (LS mean ± SEM).

VARIABLE	Control	Sham-Control	MT-Sperm
Total motility (%)	64.4±1.8^b^	67.9±1.4^a^^b^	68.4±1.3^a^
Progressive motility (%)	62.8±1.7^b^	66.3±1.4^ab^	66.9±1.3^a^
VAP (μm/s)	113.7±4.4	117.4±2.5	117.9±2.7
VSL (μm/s)	99.6±3.3^b^^#^	105.4±2.5^ab^^#^	106.1±2.6^a^
VCL (μm/s)	165.3±4.6^b^	175.0±3.0^a^	176.2±3.3^a^
ALH (μm)	6.1±0.2	6.5±0.2	6.4±0.1
BCF (Hz)	25.3±1.0	26.3±0.8	26.4±0.8
STR (%)	66.1±2.6	68.8±2.2	69.1±2.2
LIN (%)	45.3±2.1	47.1±1.9	47.3±1.9
WOB (%)	50.5±2.2	52.5±1.9	52.6±2.0

Values are LS means ± SEM based on semen samples from 4 different bulls and three replicates each.

^a,b^ means within rows with different superscripts differ (*P* < 0.05; Tukey HSD Multiple Pairwise Comparisons).

^#^*P* = 0.054. Total motility (%): percentage of moving sperm in the entire sample. Progressive motility (%): percentage of sperm that are swimming in a mostly straight line or huge circles. VAP: Average Path Velocity; VSL: Straight Line Velocity; VCL: Curvilinear Velocity; ALH: Amplitude of Lateral Head Displacement; BCF: Beat Cross Frequency; LIN: Linearity; STR: Straightness; WOB: Wobble. Control: without any supplements. Sham-Control: Ethanol in the sperm preparation medium. MT-Sperm: Melatonin in the sperm preparation medium.

Sperm motility parameters were markedly affected by the incubation period (**Table 3**). The impact of the incubation time resulted in a significantly decreased (*P* < 0.05) percentage of total motility and progressive motility spermatozoa after 120 min and 180 min of incubation. Similarly, a significant decrease (*P* < 0.05) in the LS means of BCF, VCL was observed, while the average levels for VSL and LIN were significantly increased (*P* < 0.05).

**Table 3:** Effect of incubation time (all experimental groups combined) on sperm motion parameters assessed via CASA after 30-min, 120-min, and 180-min incubation in TALP medium (LS mean ± SEM).

VARIABLE	30-min	120-min	180-min
Total motility (%)	73.1±0.9^a^	64.5±1.3^b^	63.0±1.7^b^
Progressive motility (%)	71.2±0.9^a^	63.1±1.3^b^	61.7±1.7^a^
VAP (μm/s)	113.0±2.3	120.0±3.9	116.2±3.5
VSL (μm/s)	98.6±2.1^b^	105.9±2.8^a^	106.6±3.4^a^
VCL (μm/s)	179.1±3.0^a^	172.5±3.6^a^^b^	164.9±4.3^b^
ALH (μm)	7.0±0.1^a^	6.2±0.1^b^	5.8±2.2^c^
BCF (Hz)	28.3±0.8^a^	25.4±0.9^b^	24.3±0.7^b^
STR (%)	69.4±1.9	67.1±2.5	67.5±2.7
LIN (%)	45.2±1.5^b^	46.4±2.1^a^^b^	48.3±2.2^a^
WOB (%)	51.6 ±1.6	51.3±2.2	52.7±2.3

Values are LS means ± SEM based on semen samples from 4 different bulls and three replicates each.

^a,b,c^ means within rows with different superscripts differ (*P* < 0.05; Tukey HSD Multiple Pairwise Comparisons). Total motility (%): percentage of moving sperm in the entire sample. Progressive motility (%): percentage of sperm that are swimming in a mostly straight line or huge circles. VAP: Average Path Velocity; VSL: Straight Line Velocity; VCL: Curvilinear Velocity; ALH: Amplitude of Lateral Head Displacement; BCF: Beat Cross Frequency; LIN: Linearity; STR: Straightness; WOB: Wobble.

The interaction between treatment and incubation time was not significant (*P* > 0.05) at any of the traits evaluated (**S4 Table**).

#### Cluster analysis of the detected sperm subpopulations (SPs)

The comparison of MT-Sperm with the Sham-Control group yielded only inconclusive results. Thus, a cluster analysis was made and summarized as shown in **S1 Fig**. A solution with five main sperm subpopulations (SPs; SP1 to SP5) was chosen which explained 82.9% of the variance in the dataset. A cluster was considered as main cluster, if at any combination of treatment by incubation time ≥ 5% of the spermatozoa were assigned to it. A summary of the motility descriptors for the individual cluster is provided in **S1A Fig**. No general treatment effect (*P* > 0.05; Cramer’s V: 0.02) was observed on the distributions of sperm subpopulations amongst experimental groups (**S1B Fig**). A significant overall effect of incubation time was detectable, although the effect of incubation time was estimated to be relatively small (*P* < 0.05; Cramer’s V: 0.19; **S1C Fig**). Nonetheless, the change between 30 min and 180 min incubation time was characterized by a clear decrease in spermatozoa with fast VCL, wide ALH and low LIN (SP3: 23.1% to 9.2% and SP5: 12.7% to 6.2%) and a concomitant increase in spermatozoa with moderate kinematic parameters (SP2: 32.2% to 60.1%). A stratified view of the data confirmed that the melatonin treatment did not have an effect on sperm distribution to the different subpopulations at any incubation time (**S1D Fig**).

### Sperm viability, acrosome integrity, and mitochondrial activity

Assessment of sperm quality and function by flow cytometry did not reveal significant (*P* > 0.05) effects of treatment or incubation time on the evaluated parameters (**Table 4**) and the interaction between treatment and incubation time (**S5 Table**).

**Table 4:** Effect of melatonin addition to the IVF protocol on overall sperm quality and function assessed by flow cytometry (LS mean ± SEM).

VARIABLE	Control	Sham-Control	MT-Sperm
Viable (%)	51.1±1.4	51.5±1.7	53.1±1.5
Viable, acrosome intact (%)	39.8±1.6	40.6±1.8	42.3±1.7
Viable, acrosome reacted (%)	11.3±0.4	10.8±0.4	10.9±0.4
Viable, high MMP (%)	61.4±2.0	61.2±1.9	59.5±2.0

Values are LS means ± SEM based on semen samples from 4 different bulls and three replicates each for semen assessment. Values from different incubation times, i.e. 30 min, 120 min, and 180 minutes have been combined according to the experimental groups. Control: without any supplements. Sham-Control: Ethanol in the sperm preparation medium. MT-Sperm: Melatonin in the sperm preparation medium. High MMP: high mitochondria membrane potential. No statistically significant differences with respect to the factors treatment or incubation time were observed (*P* > 0.05).

### Determination of fertilization

Chi-Square testing revealed a significant increase (*P* < 0.01) of the percentage of regular, i.e. monospermic fertilization in COCs exposed to sperm pre-treated with melatonin during the sperm preparation protocol (MT-Sperm: 66.2%) compared to that found in the Control (57.1%) and Sham-Control (43.4%) groups **(Table 5)**. Similarly, the group MT-Co-incubation had a greater proportion of regularly fertilized COCs than Sham-Control (65.6% vs. 43.4%, respectively), but not (*P* > 0.05) when compared with the Control or MT-Sperm (65.6% vs. 57.1% and 66.2%, respectively). Regular fertilization was also greater for the Control (57.1%) (*P* < 0.05) compared to Sham-Control (43.4%). On the other hand, the percentage of zygotes with polyspermy was greater (*P* < 0.01) in the control groups (Control: 16.1%, Sham Control: 13.2%) compared to that found in the melatonin groups (MT-Sperm: 9.5%, MT-Co-incubation: 9.4%).

**Table 5:** Effect of melatonin addition to the IVF protocol on *in vitro* fertilization and polyspermy rates.

Treatment Group	Zygotes (*n*)	^(+)^ Fertilization *n* (%)	Polyspermy *n* (%)
**Control**	56	32 (57.1)^b^	9 (16.1)^a^
**Sham-Control**	76	33 (43.4)^c^	10 (13.2)^a^
**MT-Sperm**	74	49 (66.2)^a^	7 (9.5)^a^
**MT-Co-incubation**	64	42 (65.6)^a^^a^	6 (9.4)^a^

^a,b,c^ Chi-Square test: Proportions within columns with different superscripts differ (*P* < 0.01). ^(+)^ Fertilization refers to normal-fertilized zygotes with the presence of two pronuclei. Polyspermy refers to those zygotes with at least three or more pronuclei identified. Control (n: 56): without any supplements. Sham-Control (n: 76): Ethanol in the sperm preparation medium. MT-Sperm (n: 74): Melatonin in the sperm preparation medium. MT-Co-incubation (n: 64): melatonin in the *in vitro* fertilization co-incubation medium. Data were collected from five IVF replicates.

### *In vitro* embryo production

Logistic regression analysis revealed similar cleavage rates (*P* > 0.05) amongst the experimental groups (Control: 73.7%, Sham-Control: 80.3%, MT-Sperm: 79.3%, MT-Co-incubation: 79.6% (**Table 6**). However, the blastocyst rate from the MT-Sperm group was greater (*P* < 0.05) than that from the Control group (39.1% vs. 54.8%). Logistic regression analysis showed that the odds ratio (OR) for blastocyst formation of COCs fertilized with sperm pre-treated with melatonin was 1.88 times more likely to become blastocysts than for COCs inseminated in the Control groups (95% confidence intervals: 1.115–3.180). Similarly, the percentage of advanced blastocysts (expanded, hatching, and hatched blastocysts) was greater (*P* < 0.05) in the MT-Sperm (33.0%) group compared to the Control group (20.9%). Moreover, the OR showed that addition of melatonin to the sperm preparation protocol made it 1.87 times more likely to produce advanced stages of the embryos than the Control group (95% confidence intervals: 1.033–3.390). Statistical analysis did not reveal differences in the blastocyst rate and the proportion of advanced embryo development in both, Control and Sham-Control group; the same was true for the MT-Sperm and MT-Co-incubation groups, respectively.

**Table 6:** Effect of melatonin addition to the IVF protocol on *in vitro* embryo developmental rates.

Treatment Group	Zygotes *n*	Cleavage *n* (%)	Blastocysts *n* (%)	Advanced bl.*n* (%)
**Control**	156	115 (73.7)	45 (39.1)^b^	24 (20.9)^b^
**Sham-Control**	147	118 (80.3)	54 (45.8)^a^^b^	35 (29.7)^a^^b^
**MT-Sperm**	145	115 (79.3)	63 (54.8)^a^	38 (33.0)^a^
**MT-Co-incubation**	152	121 (79.6)	58 (47.9)^a^^b^	33 (27.3)^a^^b^
**Average**	600	469/600 (78.2)	220/469 (46.9)	130/469 (27.7)
**Summary statistics (logistic procedure)**				
***P value***	-	NS	0.0179	0.04
**OR**	-	-	1.885	1.871
**95% CI**	-	-	1.115–3.184	1.033–3.390

^a,b^ Logistic procedure: Percentages within columns with different superscripts differ (*P* < 0.05). Blastocyst rate is considered as the proportion of cleaved zygotes that reached the blastocyst stage. Advanced bl. refers to advanced blastocysts (expanded and hatching) based on the number of cleaved zygotes. OR: Odds ratio. 95% CI: 95% confidence intervals limits for relative risks. Control (n: 156): without any supplements. Sham-Control (n: 147): Ethanol in the sperm preparation medium. MT-Sperm (n: 145): Melatonin in the sperm preparation medium. MT-Co-incubation (n: 152): melatonin in the *in vitro* fertilization co-incubation medium. Cleavage: NS: no significant (*P* > 0.05). Data were collected from six IVF replicates.

### Gene expression analysis and embryo competence of *in vitro*-derived embryos

**Fig 2:**
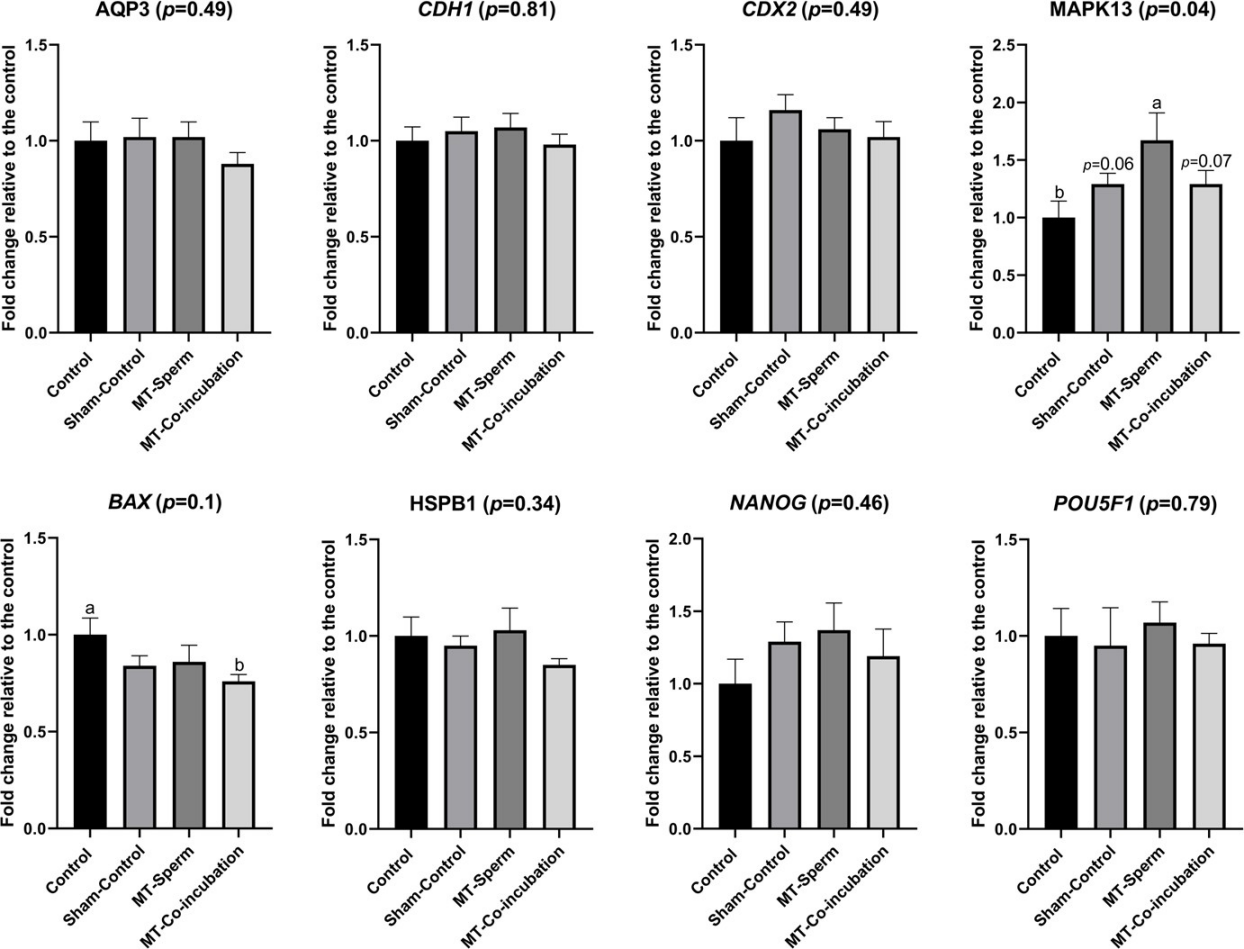
Relative mRNA abundance of genes related to embryo developmental pathways in expanded blastocysts derived from sperm treated or not with melatonin in the IVF protocol. Cadherin 1 (*CDH1*), caudal type homeobox 2 (*CDX2*), the regulator of MAP kinase activity (MAPK13), water transport gene aquaporin 3 (*AQP3*)], apoptosis regulation [BCL2 Associated X, Apoptosis Regulator (*BAX*), and Heat shock protein beta-1 (*HSPB1*)], and pluripotency [POU domain, class 5, transcription factor 1 (*POU5F1*), and the Nanog Homeobox (*NANOG*)] in the *in vitro*-derived expanded blastocysts. Data are represented as fold change relative to the control after normalizing the geometric mean from the housekeeping genes (Globin/*GAPDH*) and are means ± SEM. Different letters (a-b) indicate significant differences amongst experimental groups (*P* < 0.05). Percentages with *p* values above the SEM show statistical trends, whereas *p* values enclosed within parenthesis refer to significance level found in the statistical model for each evaluated gene.

Results from the gene expression analysis are summarized in **Fig 2**. The relative abundance of the regulator of MAP kinase activity (*MAPK13*) was significantly greater (*P* < 0.05) than observed in the Control group, but only marginally different from that found in the Sham-Control (*P* = 0.06) and MT-Co-incubation (*P* = 0.07) groups. The expression of the other genes related to developmental pathways [Cadherin 1 (*CDH1*), caudal type homeobox 2 (*CDX2*)], and water transport [aquaporin 3 (*AQP3*)] did not differ (*P* > 0.05) between treatments.

Transcript levels of the pro-apoptotic BCL2 Associated X, Apoptosis Regulator (*BAX*) gene, were lower in the MT-Co-incubation group compared to the Control group (*P* < 0.05), albeit marginal statistical significance in the model (*P* = 0.1) was observed. The mRNA abundance of the gene Heat shock protein beta-1 (*HSPB1*) did not differ amongst experimental groups (*P* > 0.05).

The relative abundance of mRNA for the pluripotency-related genes POU domain, class 5, transcription factor 1 (*POU5F1*), and Nanog homeobox (*NANOG*) was not significantly different amongst embryos derived from sperm derived from the different treatment groups (*P* > 0.05).

## Discussion

Here, we demonstrate that melatonin treatment of spermatozoa prior to or at the time of gamete coincubation improved monospermic fertilization rate and was beneficial for early embryo development up to the blastocyst stage after IVF. We also observed that melatonin treatment resulted in an increased proportion of advanced blastocysts (expanded, hatching, and hatched blastocysts), indicating that embryos derived from fertilization with melatonin-treated sperm may have produced changes at the molecular level that were reflected as enhanced fertilizing ability and partially improved embryo developmental competence.

Increased monospermic fertilization, as observed in the present study, unveils melatonin’s potential to improve current *in vitro* fertilization systems. For example, a high incidence of polyspermy is still an unresolved problem for *in vitro* embryo production in the pig [31], while in bovine IVF, it is considered as less common problem with unclear etiology [32]. Although polyspermy has been attributed to mechanisms regulated mainly by the oocyte [33], strategies implemented at the sperm level have successfully reduced its occurrence. For instance, high pre-freezing sperm dilution in porcine IVP increased monospermy, cleavage rate, and blastocyst formation [31]. Other researchers [34] found a significant reduction of polyspermic oocytes when boar spermatozoa were encapsulated and preserved in barium alginate membranes. However, it has to be taken into account, that despite numerous efforts, porcine IVF is still prone to polyspermic fertilization [35–37]. Thus, our results could serve as a practical strategy to increase monospermic fertilization in bovine and porcine IVF systems.

In a recent study using boar sperm under capacitating conditions, melatonin reduced the polyspermy rate, increased the proportion of spermatozoa binding to the zona pellucida, and regulated *in vitro* sperm capacitation (IVC) and subsequent progesterone-induced acrosome exocytosis (IVAE) via changes in the number of tail disulfide bridges [13]. The authors also found that melatonin dramatically decreased motility of capacitated sperm, with a maximal effect at 5 μM [13]. It is not clear how melatonin decreased the occurrence of polyspermy in our study. However, it is necessary to consider that regardless of *in vivo* or *in vitro* conditions, a high concentration of capacitated spermatozoa at the fertilization site is considered to be involved in an increased proportion of polyspermy [38]. Under capacitating conditions bull spermatozoa exposed to melatonin had reduced progressive motility and curvilinear velocity (VCL), inhibiting sperm capacitation [39]. Modulation of sperm capacitation by melatonin has been linked to reduced cAMP levels [40] and presumably could at least in part explain the reduced percentage of polyspermic zygotes after melatonin treatment found in our study. Other studies [41] observed that even though melatonin failed to affect sperm kinematic parameters and viability in ram spermatozoa, exposure to 100 pM melatonin increased the cleavage rate following IVF compared to the control.

Previous studies had shown beneficial effects of melatonin on sperm motility under different conditions in various species. For instance, short-term exposure *in vitro* to 1.0 mM of melatonin had a favorable impact on human sperm function by enhancing various sperm motility parameters [11]. Likewise, melatonin supplementation in bull semen extender increased kinetic parameters such as total motility, progressive motility, linearity, sperm track straightness, lateral head displacement, and also sperm viability and functionality after freezing-thawing [42]. Albeit we observed that melatonin improved some sperm kinematics traits (incl. total motility, progressive motility, VCL, and VSL) compared to the control, no significant differences were observed against the Sham control. The absence of differences between melatonin and ethanol treatments deserves further study. Possible presumptions could relate to synergistic effects between the two molecules or the involvement of independent pathways and mechanisms not investigated in the present study. Other investigators found that melatonin had dose-dependent adverse effects on sperm motility and forward progression in rats, which was partially attributed to ethanol as a solvent solution (0.5% final concentration) [43]. In our experiment, the final ethanol concentration (0.01%) did not negatively affect total sperm motility and progressive motility.

In the present experiment, sperm plasma membrane, acrosome integrity, and mitochondria membrane potential were not affected by melatonin treatment at the employed concentration (10^−11^ M) which is in contrast to the work performed previously [17]. On the contrary, it has been found that melatonin supplementation (10^−5^ and 10^−3^ M) protects sperm from ROS-associated damage and increased semen quality after freezing-thawing [44]. Similarly, other investigators observed improved cell viability, plasma membrane integrity, mitochondrial activity, acrosome integrity, reduced intracellular ROS levels, and subsequent embryo development of embryos originated from melatonin-treated spermatozoa [18].

Only one study had demonstrated beneficial effects of melatonin on post-thaw bovine sperm functionality and *in vitro* embryo development [18]. Although the *in vitro* sperm kinematics parameters were not improved in the referred experiment, sperm treatment with melatonin (10^−3^ M) resulted in an enhanced blastocyst rate [18]. Results presented in the current study indicate that oocytes fertilized with melatonin-treated sperm have an increased likelihood to become blastocysts compared to the controls. Moreover, a higher proportion of embryos reaching advanced embryo development stages (expanded, hatching, and hatched blastocysts) was also increased after melatonin treatment, indicating improved embryo competence and better embryo quality.

The gene expression analysis in our study revealed that expanded blastocysts derived from melatonin-treated sperm had increased *MAPK13* gene expression compared to the controls. Early bovine embryos critically depend on both MAP kinase signaling and the extracellular signal-regulated kinase (ERK) pathway to complete development to the blastocyst stage [45]. Recently, it was found that MAP kinase signaling was crucial for ICM differentiation via restricting epiblast cell numbers [46]. Increased epiblast precursors and decreased hypoblast precursors were observed when MAP kinase signaling was inhibited in bovine embryos [47]. Quantification of the number of cells expressing cell lineage segregation transcripts revealed an increase in the proportion of *NANOG*-positive cells while decreasing *GATA-*positive ones in the ICM of embryos cultured in the presence of a MAP kinase inhibitor [47]. *NANOG*-positive cells are destined to become the pluripotent epiblast, whereas *GATA*-positive cells likely become the differentiated hypoblast, also known as the primitive endoderm [48]. We did not measure the expression of *GATA* in the current study, nonetheless, increased *MAPK13* expression did not affect the transcription level of *NANOG*, *POU5F1* (required for *NANOG* expression in the bovine blastocyst [49], and the TE cell segregation transcript *CDX2* [50].

The MAP kinase pathway may play an essential role in keeping the balance in embryonic cell lineage segregation, which is critical to maintain a physiological number of hypoblast cells. The yolk sac, which develops from the hypoblast, is indispensable in early pregnancy in all mammals playing an essential role in histotroph digestion [revised in 51]. Some features involved in the lower competence of *in vitro*-derived embryos included a reduced number of embryonic cells, less elongated conceptus, smaller embryonic disk, compromised yolk sac development, marginal development of binucleate cells and cotyledon, and reduced placenta vascularization [51]. In the bovine, the growth of epidermal primary trophoblast cells was accelerated in medium supplemented with epidermal growth factor (EGF) via activation of RAS and phosphorylation of MAPK [52], suggesting that increased mRNA expression of the transcripts *MAPK13* observed in the embryos derived from melatonin-treated sperm could lead to enhanced embryo competence.

The enhanced fertilizing ability followed by improved embryo developmental rates and embryo competence via increased mRNA expression of *MAPK13* found in our study could be linked to the influence of melatonin on MAP kinase signaling at the sperm level that was further reflected in the embryos. Recently, it was shown that a lower level of the microRNA miR-216b in bovine spermatozoa and zygotes was associated with a higher level of K-RAS (a gene of the RAS/MAPK pathway) in two-cell embryos, which increased first cleavage rate and blastocyst cell numbers from bulls with high fertility, while the opposite was for low fertility bulls [53]. Similarly, transcriptomic profiling of buffalo spermatozoa revealed downregulation of 28 genes associated with MAPK signaling pathway bulls with low fertility compared to those with high fertility [54]. Thus the enhanced fertilization ability and embryo development observed in our study could hypothetically be linked to this pathway. The underlying molecular mechanisms of melatonin-treated sperm modulated MAPK signaling in the *in vitro-*derived blastocysts warrant further investigation. MAPK is involved in several male reproductive functions, incl- sperm motility, hyperactivation, capacitation, and acrosome reaction prior to fertilization in the female reproductive tract [55].

We hypothesize that molecular changes (not revealed in our study) after melatonin treatment could influence sperm ability to induce oocyte activation and subsequent embryo development via the described pathway mentioned above. Oocyte activation and repeated intracellular calcium increase lead to in-depth molecular changes, including resumption of meiosis, exocytosis of cortical granules (necessary for sperm block to avoid polyspermy at the zona level), cell cycle initiation, and maternal mRNA assembly [56]. Calcium oscillations induced by the sperm are decoded and interpreted by the oocyte to generate specific responses during activation and are essential to determine embryo development [57]. The acrosome reaction is Ca^2+^-dependent and crucial for regular fertilization, which depends upon phosphorylation of the MAP kinase pathway [58]. There is an association between cytosolic Ca^2+^ oscillations and intracellular level of ATP, and sperm-triggered Ca^2+^ cytosolic oscillations are transmitted to the mitochondria in the oocyte, where they directly stimulate mitochondrial activity [59]. Mitochondrial ATP production is an indispensable requirement for sustaining sperm-triggered Ca^2+^ oscillations during fertilization and is essential to support early embryo development [59, 60].

The majority of published research supports the hypothesis that melatonin is critically involved in sperm mitochondrial physiology. This assumption is based on the fact that sperm motility mainly depends on the flagellar movement of the sperm tail, which in turn relies on the ATP contents produced by the mitochondria located in the sperm mid-piece. Mitochondrial ATP production is an indispensable requirement for sustaining sperm-triggered Ca^2+^ oscillations during fertilization and is essential to support early embryo development [60]. Improved progressive sperm motility has been associated with intact mitochondrial membrane potential and consequently mitochondrial functionality [61]. Since we did not observe significant effects on mitochondrial membrane potential in the current study, further mitochondrial activity tests, such as mitochondrial volume measurements, analysis of mitochondrial permeability transition pores, and measurements of ATP contents, could provide better insight into the role of melatonin in sperm mitochondrial functionality and its effects on fertilization and early *in vitro* embryo development.

The effects of melatonin on mitochondrial function have been investigated in spermatozoa from various mammalian species, including ram [62, 63] and human [12]. A recent study reported a higher mitochondrial respiratory capacity of sperm cells by increasing ATP production via the oxidative phosphorylation pathway after melatonin treatment [63]. Melatonin was found to inhibit mitochondrial permeability transition pores and thereby prevented the release of pro-apoptotic factors into the cytoplasm, which in turn protected mitochondria from cryoinjury, promoted ATP synthesis, and improved sperm motility and viability of frozen-thawed sperm [63]. Melatonin improved mitochondrial membrane potential (Δψm), acrosomal integrity, and fertilization capacity of post/thaw sex-sorted bull sperm [22]. Furthermore, melatonin reduced mitochondria-derived ROS and rescued impaired penetration ability of human spermatozoa originating from mitochondrial dysfunction [12].

Finally, downregulation of the expression of the pro-apoptotic regulator BAX gene observed in embryos derived from melatonin-treated sperm in the current experiment agrees with a previous report in which melatonin improved embryo quality due to a lower number of apoptotic cells and an enhanced anti-apoptotic and antioxidant gene expression profile in the embryos [18]. The anti‐apoptotic and anti‐oxidative effects of melatonin may have reduced the oxidative stress on spermatozoa and enhanced bovine embryo quality [18]. The other genes analyzed in the present study were not significantly affected by melatonin treatment, including the lineage allocation and cell polarity Cadherin 1 (*CDH1*) and the water transport gene aquaporin 3 (*AQP3*).

In conclusion, adding melatonin to the sperm-preparation protocol for IVF enhanced sperm fertilization ability and embryo development *in vitro*. The current work suggests that the spermatozoa may play a more active role in regulating early embryo development fate than initially thought. Hence, further studies to explore this assumption are warranted.

## Supporting information

S1 Table Parameter settings CASA IVOS (Version 12.0 IVOS hamilton thorne bioscience, Beverly, USA). (DOCX)

S2 Table Flow cytometry settings flow cytometer settings (Gallios™, Beckman Coulter, Germany). (DOCX)

S3 Table Sperm quality and functionality parameters observed in the evaluated bulls. (DOCX)

S4 Table Effect of melatonin addition to the IVF protocol on freezing/thawed sperm motion parameters assessed through CASA after 30-min, 120-min, and 180-min incubation. (DOCX)

S5 Table Effect of melatonin addition to the IVF protocol on freezing/thawed sperm motion functionality assessed through FACS after 30-min, 120-min, and 180-min of the incubation period. (DOCX)

S1 Fig Cluster analysis of the detected sperm subpopulations (SPs). Distribution of bovine sperm subpopulations treated with or without melatonin during the post-thaw sperm preparation protocol for IVF after different incubation periods.
(DOCX)
