# Supplementary material for: Melatonin improves rate of monospermic fertilization and early embryo development in a bovine IVF system
Source: PLoS One. 2021 Sep 2;16(9):e0256701. doi: 10.1371/journal.pone.0256701 (PMC8412339; doi:10.1371/journal.pone.0256701)
Supplement: S1 Table — (DOCX) [file pone.0256701.s001.docx]

**S1 Table.** Parameter Settings CASA IVOS (Version 12.0 IVOS Hamilton Thorne Bioscience, Beverly, USA).

| Analysis Setup | Setting |
| --- | --- |
| Apply sort | 0 |
| Frames acquired | 60 |
| Frame rate | 60 Hz |
| Minimum contrast | 40 |
| Minimum cell size | 5 pixels |
| Minimum static contrast | 15 |
| Straightness (STR) Threshold | 25% |
| VAP cutoff | 5.0 μm/s |
| Progressive minimum VAP | 10.0 μm/s |
| VSL cutoff | 5 μm/s |
| Cell intensity | 70 |
| Static head size | 0.26 to 7.88 |
| Static head intensity | 0.14 to 1.99 |
| Static elongation | 10 to 98 |
| Slow cells motile | No |
| Magnification | 8.75 |
| Magnification | 8.75 |
| Video frequency | 60 |
| Bright field | No |
| LED illumination intensity | 2188 |
| IDENT illumination intensity | 2598 |
| Temperature, Set | 37.5 °C |
| Chamber depth | 10 μm |
| Chamber position | 14.5 μm |
| Chamber position B | 15.5 μm |
| Chamber position C | 16.5 μm |
| Chamber position D | 17.5 μm |
| Chamber type | Makler |
| Field selection mode | Auto |
| IDENT fluorescent mode | OFF |
| Integrating time | 1 Frame |
