# Supplementary material for: Melatonin improves rate of monospermic fertilization and early embryo development in a bovine IVF system
Source: PLoS One. 2021 Sep 2;16(9):e0256701. doi: 10.1371/journal.pone.0256701 (PMC8412339; doi:10.1371/journal.pone.0256701)
Supplement: S2 Table — (DOCX) [file pone.0256701.s002.docx]

**S2 Table.** Flow cytometer settings (Gallios™, Beckman Coulter) used for sperm stained for plasma membrane integrity with Sybr14/Propidium Iodide (PI) (LIVE/DEAD^®^Sperm Viability Kit), in combination with either acrosome integrity using peanut agglutinin (PNA-Alexa Fluor™ 647) or mitochondrial membrane potential (Mito Tracker™ Deep red FM). Abbreviations: FS: forward scatter, SS: side scatter, FL1: fluorescence channel 1, FL3: fluorescence channel 3, FL6: fluorescence channel 6.

| Sybr14/PI/PNA-Alexa Fluor™ 647 (PNA-AF647) | | | | | | |
| --- | --- | --- | --- | --- | --- | --- |
| Setting | **FS** | **SS** | | **FL1** | **FL3** | **FL6** |
| Voltage | 124 | 390 | | 688 | 421 | 730 |
| Gain | 2.0 | 10 | | 1.0 | 1.0 | 1.0 |
| Discriminator | 120 | Off | | Off | Off | Off |
| Sybr14/PI/Mito Tracker™ Deep red FM (Deep Red FM) | | | | | | |
| Setting | **FS** | **SS** | | **FL1** | **FL3** | **FL6** |
| Voltage | 124 | 213 | | 704 | 371 | 791 |
| Gain | 2.0 | 10 | | 1.0 | 1.0 | 1.0 |
| Discriminator | 120 | Off | | Off | Off | Off |
| Parameter Settings | | | | | | |
| Compensation factor (FL1 to FL3) | | | Sybr14/PI/PNA-AF647 | | | 14.6 |
|  |  |  | Sybr14/PI/Deep red FM | | | 20.9 |
| Compensation factor (FL3 to FL1) | | | Sybr14/PI/PNA-AF647 | | | 0.4 |
|  |  |  | Sybr14/PI/Deep red FM | | | 0.1 |
| Compensation factor (FL3 to FL6) | | | Sybr14/PI/PNA-AF647 | | | 0.0 |
|  |  |  | Sybr14/PI/Deep red FM | | | 0.0 |
| Compensation factor (FL6 to FL3) | | | Sybr14/PI/PNA-AF647 | | | 2.4 |
|  |  |  | Sybr14/PI/Deep red FM | | | 4.5 |
| Blue laser | | | On, Shutter Closed | | | |
| Excitation wavelength (nm) Sybr14 | | | 488 | | | |
| Excitation wavelength (nm) PI | | | 488 | | | |
| Excitation wavelength (nm) PNA-AF647 | | | 633 | | | |
| Excitation wavelength (nm) Deep red FM | | | 633 | | | |
| Detection wavelength FL1: | | | 525 nm: 40 band pass | | | |
| Detection wavelength FL3: | | | 620 nm: 30 band pass | | | |
| Detection wavelength FL6: | | | 660 nm: 20 band pass | | | |
| Sybr14 detection channel | | | FL1 | | | |
| PI detection channel | | | FL3 | | | |
| PNA-AF647 detection channel | | | FL6 | | | |
| Deep red FM detection channel | | | FL6 | | | |
| Software version | | | Gallios 1.2 | | | |
