# Supplementary material for: Melatonin improves rate of monospermic fertilization and early embryo development in a bovine IVF system
Source: PLoS One. 2021 Sep 2;16(9):e0256701. doi: 10.1371/journal.pone.0256701 (PMC8412339; doi:10.1371/journal.pone.0256701)
Supplement: S3 Table — (DOCX) [file pone.0256701.s003.docx]

**S3 Table.** Sperm quality and functionality parameters observed in the evaluated bulls.

|  | Bull | | | |
| --- | --- | --- | --- | --- |
| Variable | **1** | **2** | **3** | **4** |
| Total motility (%) | 73.9±2.0 | 73.2±1.6 | 74.3±1.6 | 71.0±2.0 |
| Progressive motility (%) | 72.0±6.0 | 71.3±4.6 | 72.1±1.5 | 69.2±1.9 |
| Viable (%) | 65.6±1.2^a^ | 60.9±2.5^ab^ | 61.9±1.2^ab^ | 54.9±2.1^b^ |
| Viable, acrosome intact (%) | 56.5±1.7^a^ | 48.8±3.1^a^ | 52.3±1.5^ab^ | 42.5±2.1^b^ |
| Viable, high MMP (%) | 64.4±0.8^b^ | 56.5±2.5^c^ | 62.7±1.2^bc^ | 77.7±1.9^a^ |

Values are the means ± SEM. ^a,b^ means among rows with different superscripts differ (*P* < 0.05); Tukey HSD Multiple Pairwise Comparisons). Total motility (%): percentage of moving sperm in the entire sample. Progressive motility (%): percentage of sperm swimming in a mostly straight line or huge circles. Viability and acrosome integrity were evaluated with a combined staining of Sybr14, propidium iodide (PI), and PNA-Alexa Fluor 647 (PNA-AF647). Viable sperm (Sybr14 positive/PI negative) were discriminated in acrosome intact (PNA-AF647 negative) and acrosome defect/reacted spermatozoa (PNA-AF647 positive). The percentage of viable sperm with a high mitochondrial transmembrane potential (high MMP) was determined with a combined staining with Sybr14, PI and MitoTracker Deep Red FM. Bull number 4 was selected for the IVF trial.
