# Supplementary material for: Melatonin improves rate of monospermic fertilization and early embryo development in a bovine IVF system
Source: PLoS One. 2021 Sep 2;16(9):e0256701. doi: 10.1371/journal.pone.0256701 (PMC8412339; doi:10.1371/journal.pone.0256701)
Supplement: S4 Table — (DOCX) [file pone.0256701.s004.docx]

**S4 Table.** Effect of melatonin addition within the IVF protocol on freezing/thawed sperm motion parameters assessed through CASA after 30-min, 120-min, and 180-min incubation.

| Variable | Control | | | Sham-Control | | | MT-Sperm | | |
| --- | --- | --- | --- | --- | --- | --- | --- | --- | --- |
|  | **30-min** | **120-min** | **180-min** | **30-min** | **120-min** | **180-min** | **30-min** | **120-min** | **180-min** |
| Total motility (%) | 70.9±3.3^abc^ | 61.6±3.3^d^ | 60.5±3.3^d^ | 73.2±3.3^ab^ | 67.6±3.3^abcd^ | 62.8±3.3^cd^ | 75.2±3.3^a^ | 64.3±3.3^bcd^ | 65.7±3.3^bcd^ |
| Progressive motility (%) | 69.0±3.2^abc^ | 60.1±3.2^cd^ | 59.3±3.2^d^ | 71.3±3.2^ab^ | 66.2±3.2^abcd^ | 61.4±3.2^cd^ | 73.2±3.2^a^ | 62.9±3.2^bcd^ | 64.5±3.2^abcd^ |
| VAP (µm/s) | 109.4±8.4 | 120.9±8.4 | 110.7±8.4 | 113.4±8.4 | 121.4±8.4 | 117.4±8.4 | 116.2±8.4 | 117.8±8.4 | 120.3±8.4 |
| VSL (µm/s) | 95.5±7.8^b^ | 102.1±7.8^ab^ | 101.3±7.8^ab^ | 99.2±7.8^ab^ | 109.4±7.8^ab^ | 107.6±7.8^ab^ | 101.2±7.8^ab^ | 106.2±7.8^ab^ | 110.9±7.8^a^ |
| VCL (µm/s) | 173.2±9.4^ab^ | 165.8±9.4^ab^ | 156.8±9.4^b^ | 179.4±9.4^a^ | 179.2±9.4^a^ | 166.6±9.4^ab^ | 184.6±9.4^a^ | 172.6±9.4^ab^ | 171.4±9.4^ab^ |
| ALH (µm) | 6.8±0.4^abc^ | 6.0±0.4^cde^ | 5.5±0.4^c^ | 7.0±0.4^ab^ | 6.5±0.4^abcd^ | 5.9±0.1^cd^ | 7.3±0.4^a^ | 6.2±0.4^bcde^ | 5.2±0.4^cde^ |
| BCF (Hz) | 27.4±2.6^abc^ | 25.0±2.6^cd^ | 23.6±2.6^d^ | 28.2±2.6^ab^ | 26.1±2.6^bcd^ | 24.6±2.6^cd^ | 29.2±2.6^a^ | 25.1±2.6^cd^ | 24.5±2.6^cd^ |
| LIN (%) | 44.1±6.2 | 45.4±6.2 | 46.5±6.2 | 45.2±6.2 | 47.7±6.2 | 48.5±6.2 | 46.2±6.2 | 46.1±6.2 | 49.7±6.2 |
| STR (%) | 67.6±7.6 | 65.7±7.6 | 65.1±7.6 | 69.4±7.6 | 69.0±7.6 | 67.9±7.6 | 71.1±7.6 | 66.6±7.6 | 69.5±7.6 |
| WOB (%) | 50.3±6.6 | 50.1±6.6 | 51.0±6.6 | 51.5±6.6 | 52.9±6.6 | 53.1±6.6 | 52.9±6.6 | 50.9±6.6 | 54.0±6.6 |

Values are the means ± SEM. ^a,b,c,d^ means among rows with different superscripts differ (*P* < 0.05); Tukey HSD Multiple Pairwise Comparisons). Total motility (%): percentage of moving sperm in the entire sample. Progressive motility (%): percentage of sperm that are swimming in a mostly straight line or huge circles. VAP: Average Path Velocity; VSL: Straight Line Velocity; VCL: Curvilinear Velocity; DAP: Distance Average Path; DSL: Distance Straight Line; DCL: Distance curvilinear; ALH: Amplitude of Lateral Head Displacement; BCF: Beat Cross Frequency; LIN: Linearity; STR: Straightness; WOB: Wobble. Control: without any supplements. Sham-Control: Ethanol in the sperm preparation medium. MT: Melatonin in the sperm preparation medium. Warning: The interaction treatment by incubation period within experimental groups was not significant in the overall statistical model (*P* > 0.05).
