# Supplementary material for: Melatonin improves rate of monospermic fertilization and early embryo development in a bovine IVF system
Source: PLoS One. 2021 Sep 2;16(9):e0256701. doi: 10.1371/journal.pone.0256701 (PMC8412339; doi:10.1371/journal.pone.0256701)
Supplement: S5 Table — (DOCX) [file pone.0256701.s005.docx]

**S5 Table.** Effect of melatonin addition within the IVF protocol on freezing/thawed sperm motion functionality assessed through FACS after 30-min, 120-min, and 180-min of the incubation period.

| Variable | Control | | | Sham-Control | | | MT-Sperm | | |
| --- | --- | --- | --- | --- | --- | --- | --- | --- | --- |
|  | **0** | **2** | **3** | **0** | **2** | **3** | **0** | **2** | **3** |
| Viable (%) | 58.3±2.0^a^ | 48.0±1.8^b^ | 47.0±2.0^b^ | 61.3±2.1^a^ | 49.9±1.8^b^ | 43.4±1.8^b^ | 62.6±2.3^a^ | 48.8±1.5^b^ | 48.4±1.8^b^ |
| Viable, high MMP | 64.4±2.9^ab^ | 61.2±3.6^abc^ | 58.5±4.0^bc^ | 65.4±2.9^ab^ | 59.8±3.2^abc^ | 58.9±3.7^abc^ | 67.5±3.0^a^ | 57.6±2.8^bc^ | 53.5±3.4^c^ |
| Viable, acrosome intact | 47.0±2.4^ab^ | 36.7±2.0^c^ | 35.8±2.6^c^ | 50.4±2.9^a^ | 38.7±2.2^bc^ | 33.4±2.2^c^ | 51.7±2.8^a^ | 37.2±1.8^c^ | 38.0±2.4^c^ |
| Viable, acrosome reacted | 11.3±0.7 | 11.3±0.7 | 11.2±0.9 | 11.1±0.7 | 11.2±0.6 | 10.0±0.9 | 10.9±0.6 | 11.3±0.5 | 10.4±0.9 |

Values are the means ± SEM. ^a,b,c^ means among rows with different superscripts differ (*P* < 0.05); Tukey HSD Multiple Pairwise Comparisons). Values are the means ± SEM. Viability and acrosome integrity were evaluated with a combined staining of Sybr14, propidium iodide (PI), and PNA-Alexa Fluor 647 (PNA-AF647). Viable sperm (Sybr14 positive/PI negative) were discriminated in acrosome intact (PNA-AF647 negative) and acrosome defect/reacted spermatozoa (PNA-AF647 positive). The percentage of viable sperm with a high mitochondrial transmembrane potential (high MMP) was determined with a combined staining with Sybr14, PI and MitoTracker Deep Red FM. Control: without any supplements. Sham-Control: Ethanol in the sperm preparation medium. MT: Melatonin in the sperm preparation medium. Warning: The interaction treatment by incubation period within experimental groups was not significant in the overall statistical model (*P* > 0.05).
