# Supplementary material for: Melatonin improves rate of monospermic fertilization and early embryo development in a bovine IVF system
Source: PLoS One. 2021 Sep 2;16(9):e0256701. doi: 10.1371/journal.pone.0256701 (PMC8412339; doi:10.1371/journal.pone.0256701)
Supplement: S1 Fig — Distribution of bovine sperm subpopulations treated with or without melatonin during the post-thaw sperm preparation protocol for IVF after different incubation periods. (DOCX) [file pone.0256701.s006.docx]

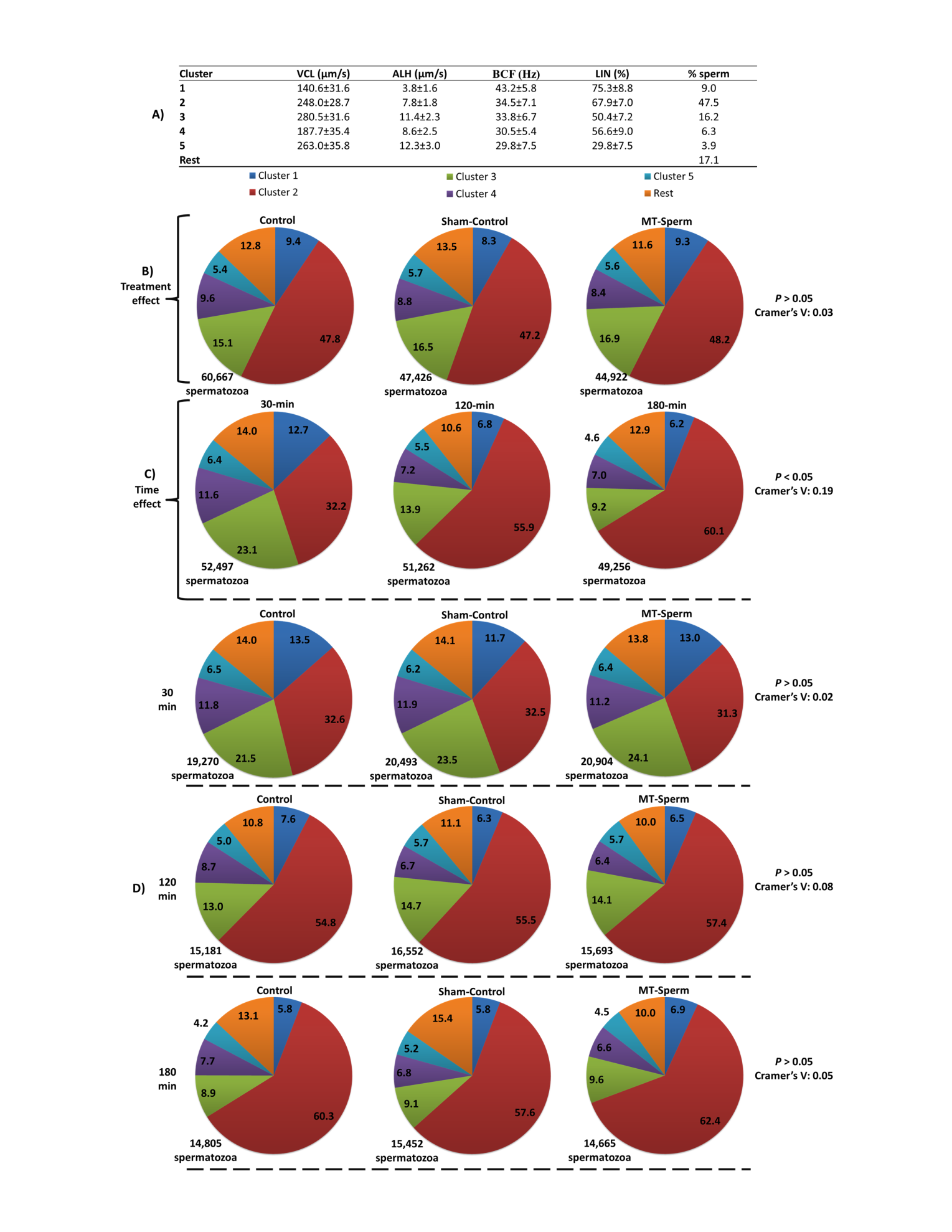


**S1 Fig. Cluster analysis of sperm kinematic parameters.** Distribution of bovine sperm subpopulations treated with or without melatonin during the post-thaw sperm preparation protocol for IVF after different incubation periods. Treatment groups: Control: standard TALP modified-medium, Sham-control: group containing ethanol in the TALP modified-medium, and MT-Sperm: melatonin added to the TALP modified-medium; times of incubation in TALP medium (30 min, 120 min, and 180 min). The analysis was based on a total of 153,015 sperm tracks from 108 samples recorded after 30, 120 and 180 minutes of incubation period. Only clusters containing more than 5% spermatozoa are displayed. A) Motility descriptors (mean ± S.D.) are given for each cluster as well as the percentage of sperm assigned to each of them. Distribution of spermatozoa to different clusters based on motility parameters to the different treatment groups (B, all time points combined), incubation period (C, all treatments combined) and a stratified view of the dataset (D). Sperm distribution to the different clusters changed significantly depending on the incubation time (*P* < 0.05). Cramer’s V (ranging from 0-1) indicates the effect size that incubation or the treatment had on the distribution of sperm to the different clusters, i.e., the size of sperm subpopulations with distinct motility patterns. For interpretation, the following definitions were used: V < 0.10 = no effect, 0.10 < V ≤ 0.30 = slight effect, 0.30 < V ≤ 0.50 = moderate effect, V > 0.50 = strong effect.
